# Reports from the Case Book of Thos. S. Powell, M. D., Sparta, Georgia

**Published:** 1856-01

**Authors:** 


### ARTICLE II.—*Reports from the Case Book of* THOS. S. POWELL, M. D., Sparta, Ga.

MESSRS. EDITORS: In your very complimentary notice of my "POCKET FORMULARY AND PHYSICIAN'S MANUEL," you say you will look to me as a regular contributor to your Journal. I am sorry that my engagements will preclude me from that pleasure; but whenever I have a spare moment, I will give you a brief history of such cases as have occurred in my practice, that I think will be of practical benefit to your numerous readers.

I hold that every man should be willing for his fellow man to profit by his experience. I hope, therefore, that your Journal will be made the channel for an extensive interchange of ideas; and every physician who has, by thought or experiment, discovered anything connected with his profession that would have a tendency to alleviate human suffering, or raise the standard of the profession in Georgia, will give your readers the benefit of it; for it has certainly been at a very low ebb in the South for several years, judging from the fact that one of the ablest Southern Medical Journals, a few years since, was on the eve of discontinuing its publication, because it was not sustained by the profession. I am happy to say, that I am informed that that most excellent Journal is now very well supported, which is some evidence that the sun of medical pride and science is rising, but is barely above the horizon yet. May it, with the aid of your Journal and the Nashville Journal (for I hope that every physician in the Southern States will take both), soon reach its meridian splendor, and shine with rays that will effectually dispel the old and superstitious idea that there is no way so good as the old beaten way of quackery and charlatanism. Then will our profession stand upon that elevated platform that God intended it. When will this be? When every medical man subscribes for four or more of the best journals in this department of science. When they read and digest all these journals contain, and when they convey to others less favored, through this medium, the benefit of the knowledge they have acquired. As Georgians are somewhat behind in their efforts to disseminate useful and practical information connected with the practice of medicine, and I, hitherto, as guilty in this respect as others, I beg leave to submit to the perusal of your readers a brief account of two more cases from my Case Book, if you should think them worthy of publication.

Case I. A young lady, aged about 23 years, was attacked, in the spring of 1853, with inflammation of the Tonsil, which her parents thought was nothing more than a severe cold. Various domestic remedies were administered; but, notwithstanding the multiplicity of remedies, the symptoms continued to increase in severity. On the fifth day, her articulation became difficult, as, also, her breathing and swallowing. The inflammation continued to spread and increase. On the sixth morning, very early, I was sent for. As the distance did not exceed two and a half miles, I suppose I reached the house in about half an hour from the time I received the message. Mr. M., her father, met me at the gate, and reported her dead. To use his language, "she had just, at that minute, choked to death." Supposing it to be a case of Tonsilitis, and that she had suffocated, I hurried in and found her supported, in a sitting position, by her mother and sister, with her eyes turned upwards, jaws distended, tongue so much swollen as to fill the mouth completely. Seeing her extreme situation, as soon as possible I pressed my thumb lancet over the tongue, and succeeded in scarifying the Tonsils. In a few moments Miss M. commenced breathing. I continued the pressure on the tongue several hours, and mopped it with a solutive Potasse Nitrus, had her feet bathed with warm water, ordered a purgative clyster applied to the throat every two hours, a warm poultice of cotton seed and mullen leaves. Six o'clock, p. m.—Symptoms improved; the swelling of the tongue and throat slightly reduced. Ordered another clyster, and the local treatment continued through the night. Nine o'clock next morning.—Great improvement; able to swallow; an evacuation from the enema. Ordered a dose of Senna and Salts; and a gargle of sugar, tea, honey, and borax to be frequently used—the poultice continued. Next morning, 10 o'clock.—Still improving; Senna and Salts had operated several times; pulse good; skin cool; tongue and tonsils very much reduced; advised the poultice—for it is a most excellent one in all affections of the throat—and the gargle to be continued, and a light diet for a few days. Dismissed the case in a few days. She was reported well.

CASE II. C. B., a fine, healthy boy, about ten years of age, was attacked February last with fever, accompanied with a cough and pains about the breast. His case was considered by his parents a severe cold, and administered some teas, in order to break the cold; but the case grew worse, and the family physician was sent for. I think he pronounced it a case of typhoid fever, and gave a small dose of Calomel and Dovers Powders every three or four hours. This treatment was continued for several days. About the tenth day the case was considered almost hopeless, and I was sent for. I reached the patient about 7 P. M.; found him in a comatose condition; skin hot; pulse 130 in the minute; tongue dry, dark, and incrusted; bowels tympanitic and inclined to diarrhea. From all I could learn from his parents of the history of the case, I pronounced it a case of inflammatory remittent fever, and the only difficulty was, that it remitted about 2 o'clock, A. M., at which time doctors prefer an undisturbed embrace in the arms of Morpheus. As the attending physician was indisposed and could not attend the case, at the urgent request of the family I took charge of the patient's treatment. I was compelled to return that night, and ordered two drops of Veratrum to be given in a spoonful of water, every two hours, the face and arms to be frequently sponged with cold water, and a stimulating Pedelemice every two hours, and at two or three o'clock, if there was any remission or abatement in the symptoms, to inject 15 grs. quinine, and 15 gts. tinct. opii, and two or three tablespoonsfull of water. Two o'clock, A. M.—Patient more rational; tongue not so dry; skin warm, but not hot; pulse 112—injected the quinine. Eight o'clock, A. M.—Patient in a slight perspiration; sleeping soundly; pulse from 100 to 104; tongue more moist. Six o'clock, P. M.—Patient has some more fever; skin pretty hot; pulse 115; the Veratrum and cold water was resorted to, as night before. Two o'clock.—I called again and found my patient in a good condition for the quinine; pulse 100; skin soft; injected 20 grs. quinine with 15 gts. tinct. opii. Eight o'clock.—Much improved; in good perspiration; pulse 90; tongue moist; drank a few spoonsfull of rice water. Six o'clock.—Still improving; pulse 88 to 90; had not been higher than 95 at any time during the day. Two o'clock, A. M.—I called again; no change since six o'clock, P. M.; repeated the Quinine injection; ordered a purgative enema next evening; gave some general directions and dismissed the case. Mr. B. came to town in a few days and reported his boy well.

I am aware that these cases are not such as to interest your more experienced readers; yet the larger class—the inexperienced—may perhaps derive some benefit from them; at least they may, from the first case reported above, learn a valuable lesson, which is of like importance to all classes of mankind. Look well before you undertake to accomplish anything; but having put your shoulders to the wheel push on, till success crowns your efforts. "Be sure you are right, then go ahead."

The second impresses upon us the great importance of doing, at the right time and in the right way, what is to be done. What care you about a ride of twelve miles, while others sleep sweetly, if by your timely efforts a fellow-creature is restored to health?—if, by this sacrifice on your part a parent's heart is gladdened? A consciousness of having done one's duty is always pleasant; but when sacrifices must be made to accomplish the end, the pleasure is richer and more lasting.

I could give a number of cases like the second, but one will suffice.

